# The Chemistry of DPPH^·^ Free Radical and Congeners

**DOI:** 10.3390/ijms22041545

**Published:** 2021-02-03

**Authors:** Petre Ionita

**Affiliations:** Faculty of Chemistry, University of Bucharest, 050663 Bucharest, Romania; petre.ionita@chimie.unibuc.ro

**Keywords:** hydrazyl, free radical, organic synthesis, spin, redox, ESR, mechanism

## Abstract

Since the discovery in 1922 of 2,2-diphenyl-1-(2,4,6-trinitrophenyl) hydrazyl stable free radical (DPPH^·^), the chemistry of such open-shell compounds has developed continuously, allowing for both theoretical and practical advances in the free radical chemistry area. This review presents the important, general and modern aspects of the chemistry of hydrazyl free radicals and the science behind it.

## 1. Introduction

**Figure 1:**
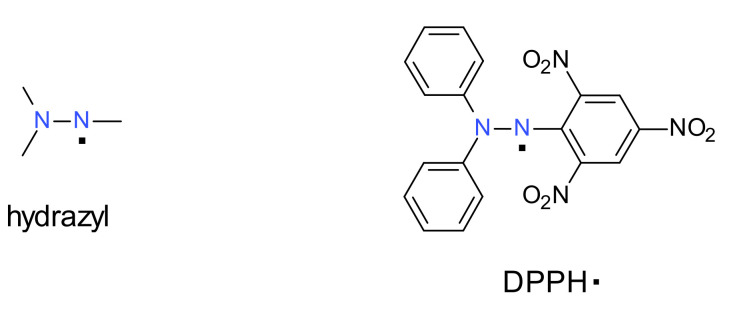
Chemical structure of DPPH^·^, a stable hydrazyl free radical.

The chemistry and physics of stable or persistent free radicals are well represented by several books [1,2,3], two of which place a greater emphasis on their organic reactions, the first one being published in 1968 [4] and the second one in 2010 [5]. However, as mentioned in the literature [6], in the most recent there is a notable absence of hydrazyl (and also aminyl) free radicals. Therefore, the first intention of this review is to highlight the most important aspects of the forgotten class of stable or persistent free radicals of hydrazyl type. This aim is focused mainly on the synthesis and physical and chemical characteristics of such free radicals, many of them derived from the most encountered stable hydrazyl (DPPH^·^, Figure 1). Although now-a-days the abbreviation DPPH is synonymous with ‘antioxidant assay’ in the literature, this subject is beyond the scope of this work.

### 1.1. Free Radicals

A free radical is a chemical entity that contains an unpaired electron (free electron) that possess a quantum-mechanical property called spin. Such an entity typically has a high reactivity due to its open-shell structure. However, nowadays there are a lot of such known compounds that are stable under usual laboratory conditions (room temperature and presence of air) [3,4,5]. Their high stability is a sum of several structural characteristics, the most important being *steric hindrance* and *conjugation*. The push–pull effect that contributes to the greater stability of several classes of such free radicals (see Section 1.3) was postulated in the 1960s [7].

### 1.2. Hydrazyls

A hydrazyl free radical contains the chemical moiety denoted in Figure 1 Left, where the dots represents the unpaired electron. The most known hydrazyl free radical is 2,2-diphenyl-1-(2,4,6-trinitrophenyl) hydrazyl, usually encountered as DPPH^·^ (the 2,4,6-trinitrophenyl fragment is also frequently named picryl).

The history of hydrazyl free radicals starts about 100 years ago, when Goldschmidt firstly observed that oxidation of triphenylhydrazine led to an intense blue color that fades rapidly [8]. Between many congeners of triphenylhydrazine, he found that the oxidation of 2,2-diphenyl-1-picrylhydrazine, a yellow compound, also gives an intense violet solution, but in this particular case the color is stable. In this way, Goldschmidt isolated in high yields the first stable hydrazyl free radical [9]. It was found that this free radical does not dimerize or react with oxygen and is stable either in solution or in solid state. However, it is still a reactive compound, active in many types of reactions, as will later be shown.

### 1.3. DPPH^·^ Free Radical

**Figure 2:**
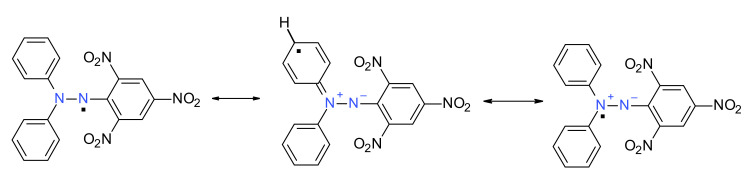
Some resonance structures of DPPH^·^ free radical.

The stability of such hydrazyl free radicals may be easily evidenced in the case of DPPH^·^. As mentioned before, steric hindrance and conjugation play a dominant role. The *push–pull effect* (as picryl moiety plays the role of the electron-acceptor part of the molecule and the diphenylamino moiety plays the role of the electron-donor part of the molecule) is clearly evidenced by the resonance structures (Figure 2) and is in accordance with Linnet theory [10]. Another important aspect that is worth remembering is the dipole moment of DPPH^·^ (4.88 D), higher than DPPH-H (3.59 D), emphasizing the importance of the polar resonance structures [11]. The N-N bond also has a higher bond order, as was directly demonstrated by the X-ray structure [12]. Due to its extreme stability, DPPH^·^ is nowadays used as a standard in Electron Spin Resonance (ESR) spectroscopy [3,13].

**Figure 3:**
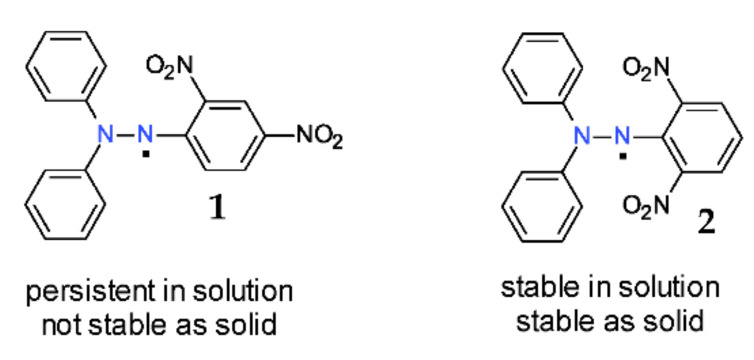
Stability of two hydrazyl isomers.

The extreme stability of the DPPH^·^ free radical is easily demonstrated by the comparison with its congeners, 2,2-diphenyl-1-(2,4-dinitrophenyl) hydrazyl **1** and 2,2-diphenyl-1-(2,6-dinitrophenyl)-hydrazyl **2** free radicals (Figure 3). The most important nitro groups are situated in the *ortho*-position with regard to the nitrogen atom; thus, between the two 2,4-dinitro and 2,6-dinitro isomers **1** and **2** (Figure 3), there is a huge difference in terms of their stability [14,15]. Accordingly, isomer **1** (2,4-dinitro) can exist only in solution (with a half-life of about 90 h) and not in solid state, while isomer **2** (2,6-dinitro) is stable both in solution and in solid state [16]. As a consequence, most of the free radicals of such type that contain any other group in position 6 (*para-)* are stable [4].

**Figure 4:**
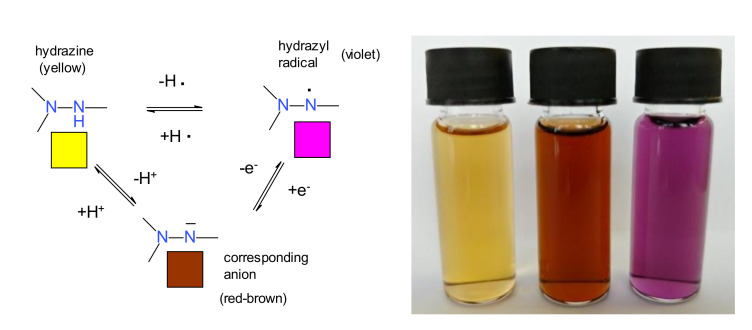
Interconversion of the parent hydrazine into a hydrazyl free radical (by oxidation) or a salt (by reaction with a base).

The hydrogen atom bound to the hydrazine group cannot only to be removed by oxidation, leading to the hydrazyl free radical, but also by a base, because it has an acidic character (this property will be discussed in detail later, in Section 4.1). Most of the hydrazyl free radicals have a violet color, while the parent hydrazines are yellow; the corresponding anion is usually red-brown, but it is possible also to be green, depending on the substituents. All these acid-base or redox processes are reversible, as shown in Figure 4 [17].

To make easier the work with such structures, in this paper a free radical will be indicated by numbers (i.e., **1**), while the parent hydrazine will be noted as **1-H**; similarly, the parent hydrazine of DPPH^·^ free radical will be noted as DPPH-H.

### 1.4. ESR Spectroscopy

A straightforward way to obtain important structural information about free radicals’ structure is to use the Electron Spin Resonance (ESR) spectroscopy (named also Electron Paramagnetic Resonance—EPR)—a technique which is applicable to free radicals, and generally speaking, to any compound or material that contains unpaired electrons. In solid state, the ESR spectrum of DPPH^·^ shows a single line, while in an appropriate solvent the ESR spectrum consists in five lines almost equidistant, with relative intensities 1:2:3:2:1, that mainly correspond to two equivalent nitrogen nuclei, with the hyperfine coupling constants *a*_*N*1_ = *a*_*N*2_ = 9 Gauss (G). However, even early theoretical calculation [18] as well as detailed analysis [19] of the ESR spectrum showed that these values are closer to the values of *a*_*N*1_ = 9.35 G and *a*_*N*2_ = 7.85 G (the higher value being associated with the *N*-picryl atom). Replacement of ^14^*N* with the ^15^*N* isotope clearly confirmed this [20,21,22]. Table 1 shows several congeners of DPPH^·^ free radicals and their ESR characteristics [4].

These hyperfine coupling constants of the two nitrogen nuclei cannot be used to assess the stability of the corresponding free radicals; for example, in the case of the 2,4-dinitro and 2,6-dinitro isomers (**1** and **2** Figure 3), these values, even they are different, cannot explain the stability of free radicals. The lowest *a_N_*_2_ value of 5.8 G is recorded for the carbazole derivative (**11**, Table 1), a stable compound like DPPH^·^ [23]. This radical has an ESR spectrum with seven lines and relative intensities of 1:1:2:1:2:1:1, demonstrating a high interaction of the free electron with the picryl ring, similar to 2,2-(*p*-dinitrophenyl)-1-picrylhydrazyl **10**.

## 2. Ways of Synthesis of Some Hydrazyls and Other Typical Reactions

### 2.1. Synthesis of DPPH^·^ and Relatives

**Figure 5:**
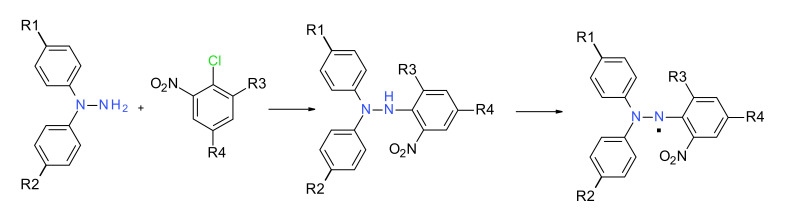
Typical synthesis of hydrazyl free radicals.

Hydrazyl free radicals are easily obtained by the oxidation of the corresponding hydrazines with lead dioxide, lead tetraacetate, silver oxide or potassium permanganate (Figure 5). Such reactions are conducted in a non-polar solvent, such as dichloromethane (DCM) or benzene, and affords by simple filtration the desired radical in almost quantitative yields [24]. A complicated oxidation mechanism is possible [25]. The starting hydrazines are easily obtained by direct coupling reaction (Figure 5). In this way, most of the compounds from Table 1 can be obtained, with the exception of the *p*-nitrophenyl derivatives **9** and **10** (in these cases no hydrazines are available by synthesis).

Many other hydrazyl persistent or stable free radicals can be obtained starting from easily functionalized derivatives, such as, for example, those that contain a carboxyl or sulfono group (such as compounds **3** or **5**, Table 1); starting from these derivatives, hydrazyls that are derivatized with interesting motifs (such as antipyrine, valine, crown-ether, etc.) were usually obtained in a single step with high yields [26,27].

### 2.2. Reaction with Nitrogen Dioxide

**Figure 6:**
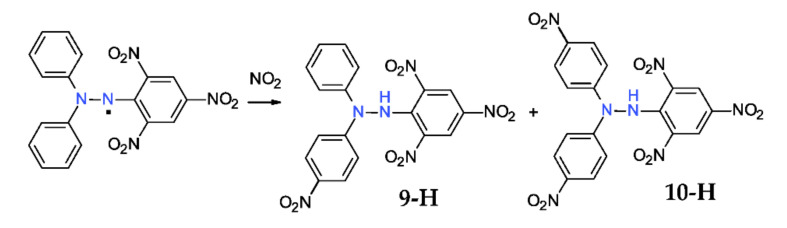
Reaction of DPPH^·^ with nitrogen dioxide.

Nitrogen dioxide is also a stable free radical and it instantly reacts with DPPH^·^**,** affording a mixture of mono- and dinitro-derivatives (**9-H** and **10-H**, Figure 6) [28]. However, it is possible to obtain selectively each one, with up to 90% yields, using a different strategy: for the mono-nitroderivative **9-H**, DPPH^·^ reacted with solid sodium nitrite in the presence of a crown-ether, using DCM as solvent [29], while for the di-nitroderivative **10-H**, DPPH^·^ reacted in a biphasic system DCM-water with nitrous acid (in fact, an acidified solution of sodium nitrite [30]). ^15^*N*-Substituted compounds can be also obtained using these approaches [31].

### 2.3. Reaction with Halogens

**Figure 7:**
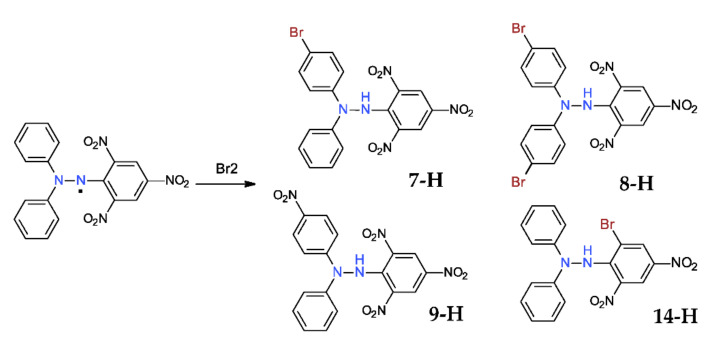
Reaction of DPPH^·^ with bromine.

Halogens react quickly with the DPPH^·^, following a radical mechanism (chlorine and bromine act similarly; fluorine gives a violent reaction, while iodine practically does not react) [32,33]. In this way, the mono- and di-bromo-derivatives (**7-H** and **8-H**, Figure 7) can be obtained, in a mixture that requires separation (or better, they can be obtained individually starting from the corresponding halogeno-diphenylhydrazynes, following the synthetic procedure from the Section 2.1).

An interesting aspect is the formation of the *p*-nitro-derivative **9-H** together with the bromo-substituted picryl **14-H** (similar reaction with chlorine was analyzed recently) [34]. The mechanism of reaction involves an *ipso*-substitution of a nitro-group that is released as nitrogen dioxide that is further captured by another hydrazyl molecule with the formation of the *p*-nitrophenyl-derivative (as mentioned before).

### 2.4. Reaction with Hydroxyl Radical

**Figure 8:**
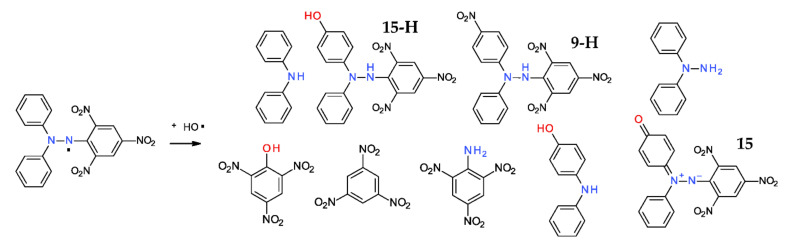
Reaction of DPPH^·^ with hydroxyl free radical, showing a wide range of decomposition products.

Hydroxyl radical is an extremely reactive, non-selective species, used even for the destruction of organic molecules in water decontamination processes [35]. Reaction of the DPPH^·^ with the hydroxyl radical yields a plethora or compounds (Figure 8), formed mainly by hydroxylation/decomposition of the starting material [36,37]. The reaction also yields the formation of the *p*-nitrophenyl-derivative **9-H**, together with the hydroxyl correspondent **15-H**, as well the oxidized betaine counterpart **15** (Figure 8).

### 2.5. Reaction with Hydroxylamine or Potassium Cyanide

**Figure 9:**
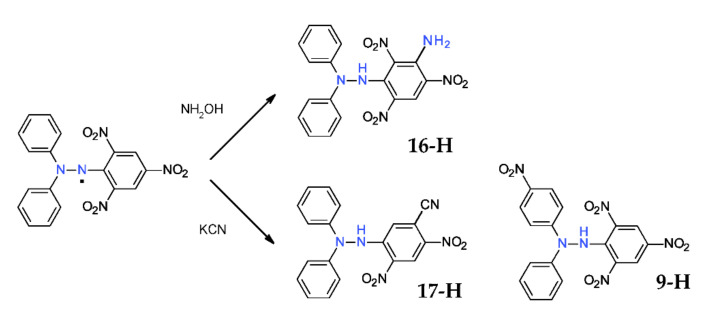
Reaction of DPPH^·^ with hydroxylamine and KCN.

Very interestingly, reaction of DPPH^·^ with hydroxylamine in basic media or with potassium cyanide does not led to the presumably *p*-phenyl-derivatives, but to compounds that are derivatized on the picryl ring (Figure 9). The formation of such derivatives follows a quite different mechanism, as will be shown in the next chapter [38,39].

### 2.6. Reaction with Syringaldehyde

**Figure 10:**
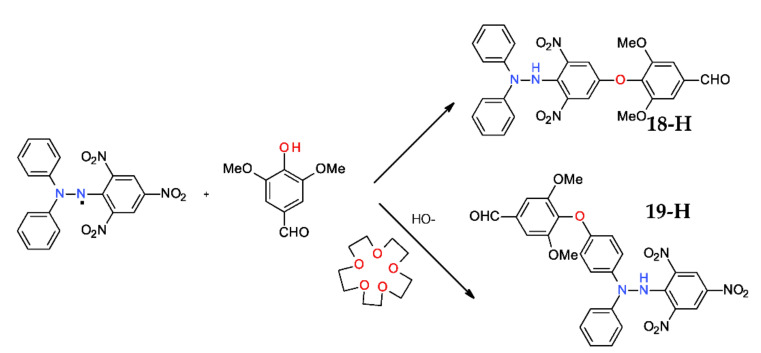
Reaction of DPPH^·^ with syringaldehide.

A more intriguing reaction takse place with the phenol derivative from Figure 10 [40]. Thus, using the plain syringaldehyde, the upper compound **18-H** (picryl substitution) is obtained in 11% yields, while, for the same reaction performed in a basic media and in the presence of a crown ether, a different compound, **19-H** (*p*-phenyl substituted), is obtained in 42% yields.

Literature data showed only a single reference in a similar case [41], in which the phenol derivative is replaced by a thiol congener. Mechanistic exploration of the reaction led to several conclusions: (i) both compounds are obtained via a radical mechanism; (ii) the main difference resides in the induced polarization of the hydrazyl free radical by the supramolecular complex formed by crown-ether, thus inducing the linking of the syringic moiety at the electron-deficient end of the hydrazyl free radical; and (iii) replacement of a nitro group by the syringaldehyde moiety is an *ipso*-substitution.

### 2.7. Betaines Derived from DPPH^·^

**Figure 11:**
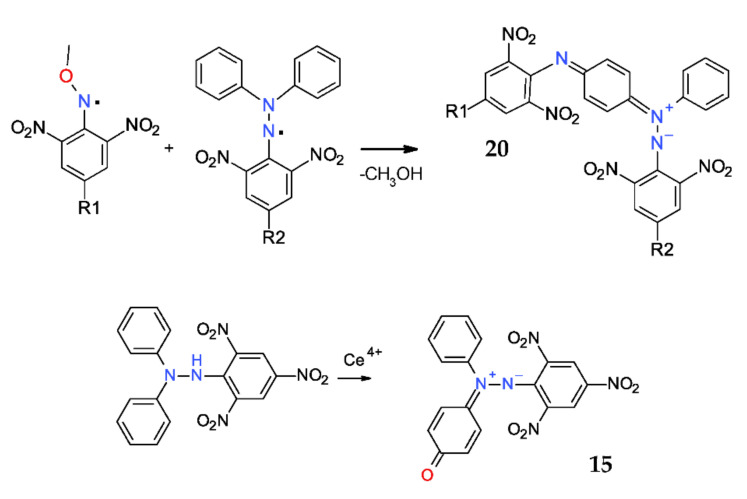
Synthesis of betaines **15** and **20.**

An astonishing reaction of the DPPH^·^ free radical was found to be with methoxyaminyl persistent free radicals (Figure 11, up), which led to a highly colored diazenium betaines **20**. Thus, in the case of R_1_ = R_2_ = NO_2_, the yields were 80%, while in the case of R_1_ = R_2_ = CN, the yields were only 54% [42,43]. The diazenium betaine **20** (R_1_ = R_2_ = NO_2_) was fully characterized by IR, MS, NMR, ESR, and most importantly, by single-crystal X-ray diffractometry [44]. The N–N bond length is similar with that observed in the case of DPPH^·^ [45]; thus, in compound **20**, the N-N bond length is 1.337 A, while the reported values for DPPH are 1.352 A (for crystals obtained from ether) and 1.321 A (for crystals obtained from CS_2_). Besides, both picryl-*N* bonds are rather short, but as expected, the *C–N* bond length for the phenyl-*N* bond clearly indicates a single bond. The *p*-phenylene group has a genuine quinonoid structure with shorter and longer bonds, thus confirming their betaine form.

Another interesting case is the betaine **15**, which was noticed as traces in the reaction of DPPH^·^ with hydroxyl radical [36]. Remarkably, the oxidation of DPPH^·^ or of the corresponding hydrazine DPPH-H with cerium sulfate affords directly the betaine **15** in a very high yields (90%) [46]. Single-crystal structure was also measured by X-ray diffractometry [47].

**Figure 12:**
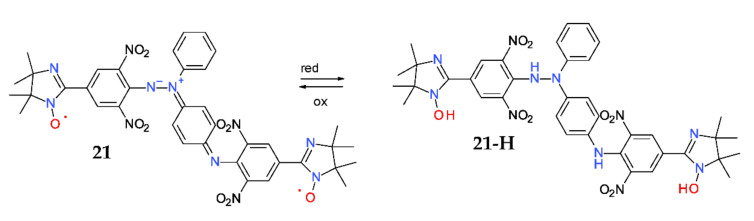
Reversible oxidation or reduction of betaine **21.**

Such betaines can be easily reduced with several reagents, such as ascorbic acid or sodium ascorbate, yielding their reduced counterparts. The process is accompanied by a dramatic color change, from blue to yellow (conversely, the reduced counterparts can be re-oxidized to the starting betaine, thus showing a reversible process). More complicated structures can be obtained, such is the diradical-betaine **21**; of course, it can be reduced as usual and the process is accompanied by color change, from blue to yellow (Figure 12) [48].

### 2.8. Dihydrazyls

**Figure 13:**
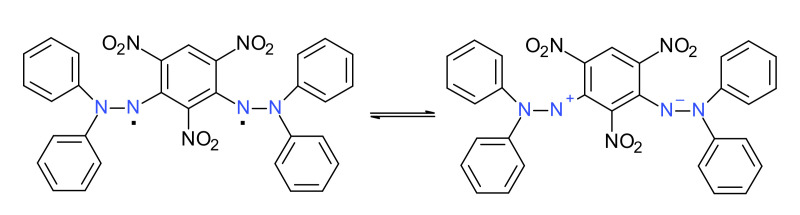
A dihydrazyl and its zwitterionic form.

Some literature data showed the synthesis of several dihydrazyls starting from their corresponding hydrazines, following the common route of oxidation [49,50]. Figure 13 shows an interesting case (in a way corresponding to the earlier presented betaine cases). Thus, oxidation of the parent hydrazine initially gave a deep-colored solution with a strong ESR signal and hyperfine coupling values very close to those of DPPH^·^, but this vanished over time, suggesting that the stable form is ionic (zwitterionic) [51].

**Figure 14:**
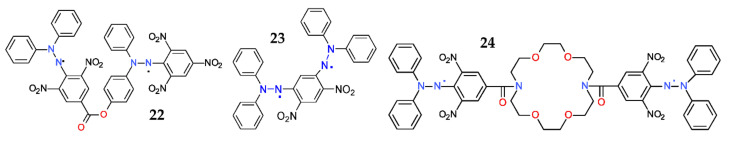
Structure of some dihydrazyls (homo-diradicals).

Other, more recent, examples of dihydrazys are shown in Figure 14. All these dihydrazyl free radicals seem to have high stability. However, there ESR spectra are quite different. Thus, for **22**, there is a strong interaction between the two unpaired electrons, showing the values of hyperfine coupling constants of about *a*_*N*1_ = *a*_*N*2_ = *a*_*N*3_ = *a*_*N*4_ = 4.3 G, meaning that there is an interaction of the spin with four equivalent nitrogen nuclei). In the case of the other two dihydrazyls **23** and **24**, the ESR spectra look very close to a simple hydrazyl free radical, with *a*_*N*1_ = *a*_*N*2_ = 9 G. Such behavior is also known in the case of nitroxide, nitronyl- or imino-nitroxide free radicals [52,53,54].

The magnetic properties of compound **22** has been also investigated in the temperature range of 2–300 K, under an applied magnetic field of 5 kG, suggesting dominant antiferromagnetic interactions. Carbazole derivatives were also prepared [55].

**Figure 15:**
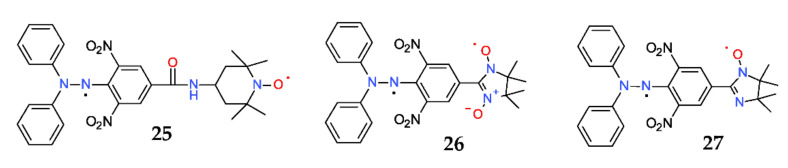
Structure of some hetero-diradicals.

Besides dihydrazyls, literature data showed another type of hydrazyl diradicals, in which the other spin is coming from a different radical moiety, such is a nitroxide free radical (if we can call dihydrazyl a homo-diradical, structures of diradicals from Figure 15 can be called hetero-diradicals). Therefore, **25** is a hydrazyl-nitroxide diradical, **26** is a hydrazyl-nitronyl-nitroxide diradical, while **27** is a hydrazyl-imino-nitroxide diradical [56].

## 3. Reaction Mechanisms

**Figure 16:**
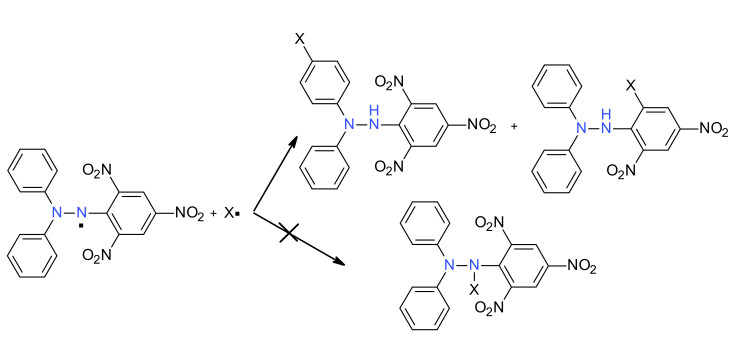
Reaction of DPPH^·^ with a free radical X**^.^**.

As was seen in all examples presented before, there are two main mechanisms that contribute to the derivatization of the DPPH^·^ molecule [57]. The first one, and the most encountered, is a free radical mechanism; thus, in a radical + radical coupling reaction, usually the *p*-phenyl adduct is obtained. However, there is also possible radical *ipso*-substitution on the picryl ring. It is also worth noting that no *N*-hydrazyl adduct was noticed in the literature until now, although some earlier data have reported such compounds (but later closer inspection of the results showed the mistake) [58]. As a result, this type of mechanism can be represented as shown in Figure 16.

**Figure 17:**
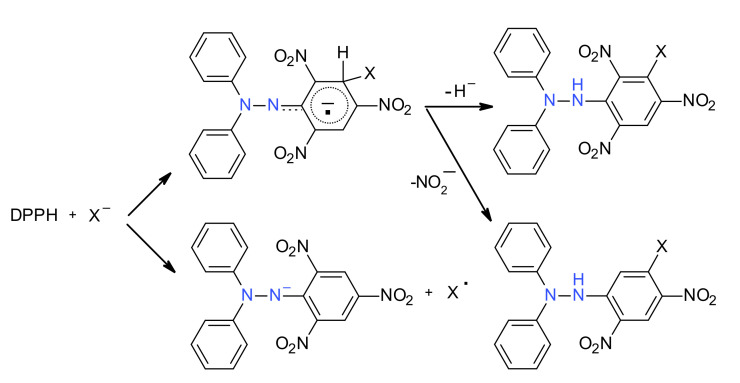
Reaction of DPPH^·^ with a nucleofile X**^−^.**

The second type of mechanism, found in some other cases, is a nucleophilic substitution. Nevertheless, after this step, radical processes may occur, following the previous mentioned mechanism. This ionic mechanism is represented in Figure 17.

The main question that arises is how can one presume the correct way that the DPPH^·^ acts. It was showed that a possible mode is to compare the redox potential of the hydrazyl radical with the redox potential of the substrate [59]. If the hydrazyl radical can oxidize the substrate, a short-lived radical is generated, and this can be captured by a radical + radical coupling reaction, leading mainly to the *p*-phenyl derivative (Figure 16). If the oxidation potential of the hydrazyl radical cannot pass the redox potential of the substrate, there are two possibilities: (i) no reaction takes place, or (ii) a nucleoplilic attack on the picryl ring occurs (Figure 17). Moreover, the correct attribution of a specific way of reaction might be difficult, as in both types of mechanism the *p*-nitrophenyl derivative of DPPH^·^ is formed. Measuring (or knowing from literature) the oxidation potential of reactants can be of real help.

## 4. Application of Hydrazyls

### 4.1. Acid-Base and Redox Processes

It was shown before that mainly the *p*-substituents on the phenyl ring has the most influence on the oxidation capacity of the DPPH^·^ congeners, as well as on their acidity (for parent hydrazines). Concerted electron–proton transfer may occur [60]. For example, each supplementary *p*-nitro group increases the oxidation potential of the free radicals by 0.1–0.2 V, while, for parent hydrazines, each group lowers the pK_a_ value by about 1 unit. This means that hydrazyl free radicals become stronger oxidants, whereas hydrazines become stronger acids. Table 2 shows these values.

All these redox or acid-base processes can be followed by color change [62]. Table 2 also compiles the wavelength values where hydrazyl free radicals, their parent hydrazines or the corresponding anions have the maximum absorption. One important aspect that can be easily evaluated, knowing the pK_a_ and E_ox_ values, is the *bond dissociation energy*- BDE for the N-H bond. This can be evaluated following the Equation (1) [63].

BDE = 1.37 pK_a_ + 23.06 E_ox_ + 56 (1)

As a remark on Table 2, it can be concluded that all the BDE values are closer to 75 kcal/mol and this is due to the fact that values of pK_a_ and E_ox_ compensate each other (DPPH has the literature BDE value ~75–80 kcal/mol [64]; a revised and accurate value was reported as 79 kcal/mol [65]). These BDE values can be regarded as an important tool in elucidation of the H-abstraction mechanism of DPPH congeners. As mentioned by Bordweel [63], most substituents on DPPH congeners will play a *dual role* in affecting the stability of such radicals, inducing *both* stabilization by the delocalizing property and at the same time destabilization by the electron-withdrawing ability. Recently, an electrografting method employing diazonium chemistry was used for the isolation of the aryl radical/DPPH coupling product [66].

### 4.2. Generators of Short-Lived Radicals

The oxidant capacity of hydrazyl free radicals can be used in the generation of short-lived radicals. It was stated before that DPPH^·^ can abstract one electron from an anion X^−^ yielding the radical X^.^ (Figure 17). In a similar way, DPPH^·^ can abstract one hydrogen atom from other compounds, yielding again unstable free radicals (for example, from phenols, Figure 10). Such processes were used in the generation of several radicals of different types, *O*-, *N*-, *S*, *C-*, or *P*-centered [67]. These short-lived radicals are best evidenced by the ESR spin-trapping technique that uses a diamagnetic unsaturated compound that reacts with the short-lived free radical forming a persistent nitroxide, with a half-life of minutes-hours. These compounds are called spin-traps, and most of them are nitrones or nitroso derivatives [68].

**Figure 18:**
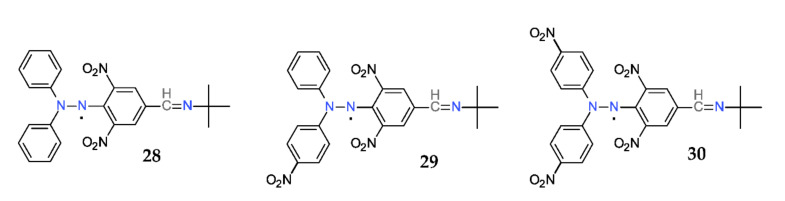
Hybrid hydrazyl-nitrones.

Because the spin-trapping process requires both the presence of a short-lived free radical and a spin-trap, and also because it is a necessary a system that generates those short-lived free radicals, a step-forwarding idea was to design hybrid molecules that contain a hydrazyl and a nitrone moiety covalently linked. Such compounds (**28–30**) are shown in Figure 18 [61].

These compounds have the great advantage of finding direct applications as sensors or probes in ESR spectroscopy, as simultaneous generators and traps for short-lived radicals. The so-called DPPH method for the total antioxidant capacity measurement is one of the most used [69,70].

## 5. Conclusions and Outlook

Besides all these general and particular aspects, hydrazyl free radicals are still a particular domain in chemistry that contains unexplored fields. Recently, DPPH^·^ found applications in very different areas, such as catalysis [71,72]; it may also may find similar applications in organic battery technologies [73,74]. Another possibility of employing hydrazyl radicals and their congeners is related to the structure of betaines **15** and **20** to exist as diradicals (similar to Thiele, Tschitschibabin or Muller hydrocarbons) [75,76,77]. Although most recent literature data is about the use of DPPH free radical as scavenger in antioxidant measurements [69,70,78], there is a lot of room to develop new compounds and processes involving such stable open-shell structures, as their multifunctionality [61,79] provides real working opportunities. Along with the well-known class of nitroxide free radicals [80], hydrazyl radicals stabilized by the captodative effect [81] will fulfill and complete novel and important possibilities. Future work will bring out more interesting and unexpected results.

## Figures and Tables

**Table 1:** Electron Spin Resonance (ESR) coupling constants (G) for several hydrazyl free radicals.

Compound	Substituents			Coupling Constants	
	R	R′	R″	*a*_*N*2_	*a*_*N*1_
DPPH^·^	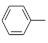	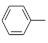	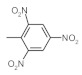	8.03	9.65
**1**	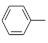	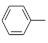	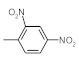	6.74	9.49
**2**	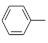	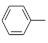	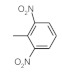	7.16	10.38
**3**	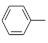	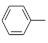	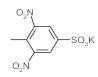	7.19	9.98
**4**	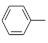	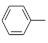	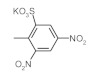	6.76	10.91
**5**	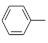	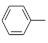	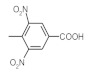	7.62	9.53
**6**	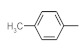	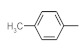	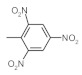	7.6	9.81
**7**	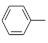	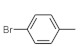	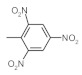	7.89	9.74
**8**	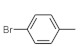	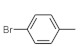	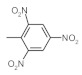	7.51	9.87
**9**	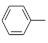	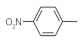	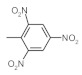	6.95	10.43
**10**	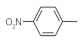	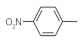	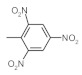	6.66	10.78
**11**	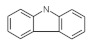		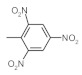	5.8	10.2
**12**	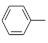	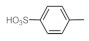	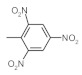	9	9
**13**	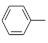	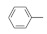	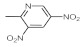	8.27	8.27

**Table 2:** λ_max_ (nm), pK_a_, E_ox_ (V) and bond dissociation energy (BDE) values (kcal/mol) for selected compounds [17,27,39,46,56,61].

Compound	λradical	λanion	λhydrazine	pKa	Eox	BDE
DPPH	518	424	322	8.54	0.30	75.3
**1**	512	383	337	11.3	0.13	74.5
**9**	500	505	352	7.35	0.49	-
**10**	500	485	352	6.49	0.60	-
**12**	524	413	353	8.20	0.34	82
**13**	506	441	363	10.7	0.25	76.4
**23**	500	365	346	11.1	0.24	76.3
**25**	524	655	324	12.5	0.87	76.81
**26**	517	495	330	11.6	1.17	76.96
**27**	517	492	332	10.9	1.23	70.67
**28**	486	620	392	7.82	0.173	75.45
**29**	507	645	404	8.07	0.107	75.08
**30**	510	654	482	8.33	0.130	75.30
